# Association between AST/ALT ratio and the risk of gestational diabetes mellitus in Korean pregnant women

**DOI:** 10.1371/journal.pone.0331484

**Published:** 2025-08-29

**Authors:** Dongqian Yang, Yuqin Shen, Rong Shuai

**Affiliations:** 1 Department of Laboratory Medicine, Changde Hospital, Xiangya School of Medicine, Central South University (The first people’s hospital of Changde city), Changde, Hunan, China; 2 Department of Obstetrics, Changde Hospital, Xiangya School of Medicine, Central South University (The first people’s hospital of Changde city), Changde, Hunan, China; University of Montenegro-Faculty of Medicine, MONTENEGRO

## Abstract

**Background:**

The aspartate aminotransferase to alanine aminotransferase (AST/ALT) ratio is associated with insulin resistance (IR) and diabetes mellitus, but its association with gestational diabetes mellitus (GDM) has been less well-studied.

**Objective:**

Our study aimed to investigate whether the AST/ALT ratio is associated with GDM in a population of pregnant Korean women.

**Methods:**

The study was a secondary analysis of a prospective cohort study. It included 623 pregnant women who were at 10–14 weeks of gestation between November 2014 and September 2016. We downloaded and analyzed the data in October 2024. A total of 575 singleton pregnant women were included after excluding missing variables. All participants were followed up until the birth of their children. AST/ALT ratio was obtained by dividing AST by ALT. AST/ALT ratio was used as a continuous variable, and a quartile categorical variable was used for analysis. Logistic regression models were used to estimate the relationship between AST/ALT ratio and GDM. Subgroup and sensitivity analyses were conducted to explore the stability of this relationship. Restricted cubic spline (RCS) curve fitting was employed to investigate potential non-linear associations.

**Results:**

Pregnant women were stratified into quartiles based on their AST/ALT ratios, with the following cutoff values: Q1 (<1.167), Q2 (1.167–1.499), Q3 (1.5–1.818), and Q4 (>1.818). The overall mean AST/ALT ratio across all participants was 1.6 ± 1.0. After adjusting for confounders, the AST/ALT ratio was negatively associated with GDM (OR=0.45, 95% CI: 0.2–0.99), with results confirmed by sensitivity and subgroup analyses.

**Conclusion:**

This study demonstrated that a lower AST/ALT ratio in early pregnancy was independently and negatively associated with the risk of GDM. The AST/ALT ratio may serve as a potential early biomarker to identify pregnant women at higher risk of GDM, contributing to targeted preventive strategies.

## Introduction

Gestational diabetes mellitus (GDM) is characterized by the initial manifestation of impaired glucose tolerance during pregnancy in women with no prior history of diabetes. Epidemiological data indicate that GDM affects approximately 15% of pregnant women globally [1], while the reported incidence in Korea is slightly lower at 11.1% [2]. As a complication of pregnancy, GDM has been associated with various adverse outcomes, including maternal retinopathy, renal damage, and psychological disorders, as well as adverse pregnancy outcomes such as gestational hypertension and macrosomia [3,4]. Furthermore, the offspring of pregnant women with GDM have an increased risk of developing endocrine disorders [5], which can impose a significant social and economic burden on healthcare systems. GDM is typically diagnosed during the second or third trimester of pregnancy, around the 24–28 gestational weeks [6]. However, if a diagnosis is made at this time, there is a possibility that the mother and fetus may have been compromised to some extent. Therefore, it is imperative to identify pregnant women at risk of GDM at an early stage to reduce adverse effects and prevent intergenerational transmission of metabolic diseases.

The liver is a major metabolic site that regulates the body’s sugar, fat, and protein levels, and its abnormal function is closely linked to the development of insulin resistance (IR) and type 2 diabetes [7,8]. A series of serum enzymes are often measured to reflect the degree of liver injury, such as aspartate aminotransferase (AST) and alanine aminotransferase (ALT) are the most common markers of liver injury, which are released from the cells into the bloodstream after hepatocyte injury. The AST/ALT ratio is also known as the De Ritis ratio, named after De Ritis, and is currently used as an auxiliary diagnostic indicator of chronic liver injury [9]. Recent studies have shown that the AST/ALT ratio is associated with the occurrence and development of other diseases. For example, in urological diseases, an elevated AST/ALT ratio threatens the prognosis of surgery in patients with uroepithelial carcinoma [10]. Similarly, patients with Kawasaki disease have a linear relationship between their serum AST/ALT ratio and their coronary artery lesions (CALs), which can be used as a risk factor to assess the risk of their disease. In addition, people with high AST/ALT ratios have a relatively increased risk of cardiovascular disease, hematological disorders, and heart disease due to respiratory viral infections [11–13]. Therefore, we believe that this ratio is correlated with inflammation, metabolism, and oxidative stress.

The AST/ALT ratio shows several advantages over traditional predictors of GDM. Firstly, AST/ALT is a routine part of prenatal screening and is readily available without additional testing infrastructure. Second, unlike postprandial fluctuating blood glucose indicators, the AST/ALT ratio reflects chronic subclinical metabolic dysfunction, potentially identifying those at risk before clinical hyperglycaemia occurs [14]. Third, it reflects insulin resistance, inflammation and oxidative stress [15,16].

A high baseline AST/ALT ratio often predicts the occurrence and progression of some diseases and poor outcomes. However, in studies of diabetes, the AST/ALT ratio is negatively correlated with the occurrence and progression of diabetes [17,18]. It has been shown that the ALT/AST ratio is positively associated with insulin resistance in the Korean population [19]. GDM and type 2 diabetes mellitus have similar pathogenesis, suggesting that the AST/ALT ratio may be associated with the occurrence of GDM. This study aimed to elucidate the association between AST/ALT ratio and gestational diabetes mellitus in a Korean pregnant population.

## Methods

### Data source

Data for our study came from the original Lee et al. study (NCT 02276144) ‘Fatty Liver Disease in Pregnancy’ registry. This data can be downloaded from PLoS ONE (https://journals.plos.org/plosone) [20]. They initially recruited singleton pregnant women who underwent antenatal check-ups before 14 weeks of gestation at Seoul Women’s Hospital in Incheon and Seoul Metropolitan Government Seoul National University Boro Ex-Medical Centre between November 2014 and September 2016 to determine the risk of NAFLD on pregnancy outcomes. Throughout the study, each pregnant participant signed an informed consent form and strictly adhered to the Declaration of Helsinki.

### Study population

**Fig 1:**
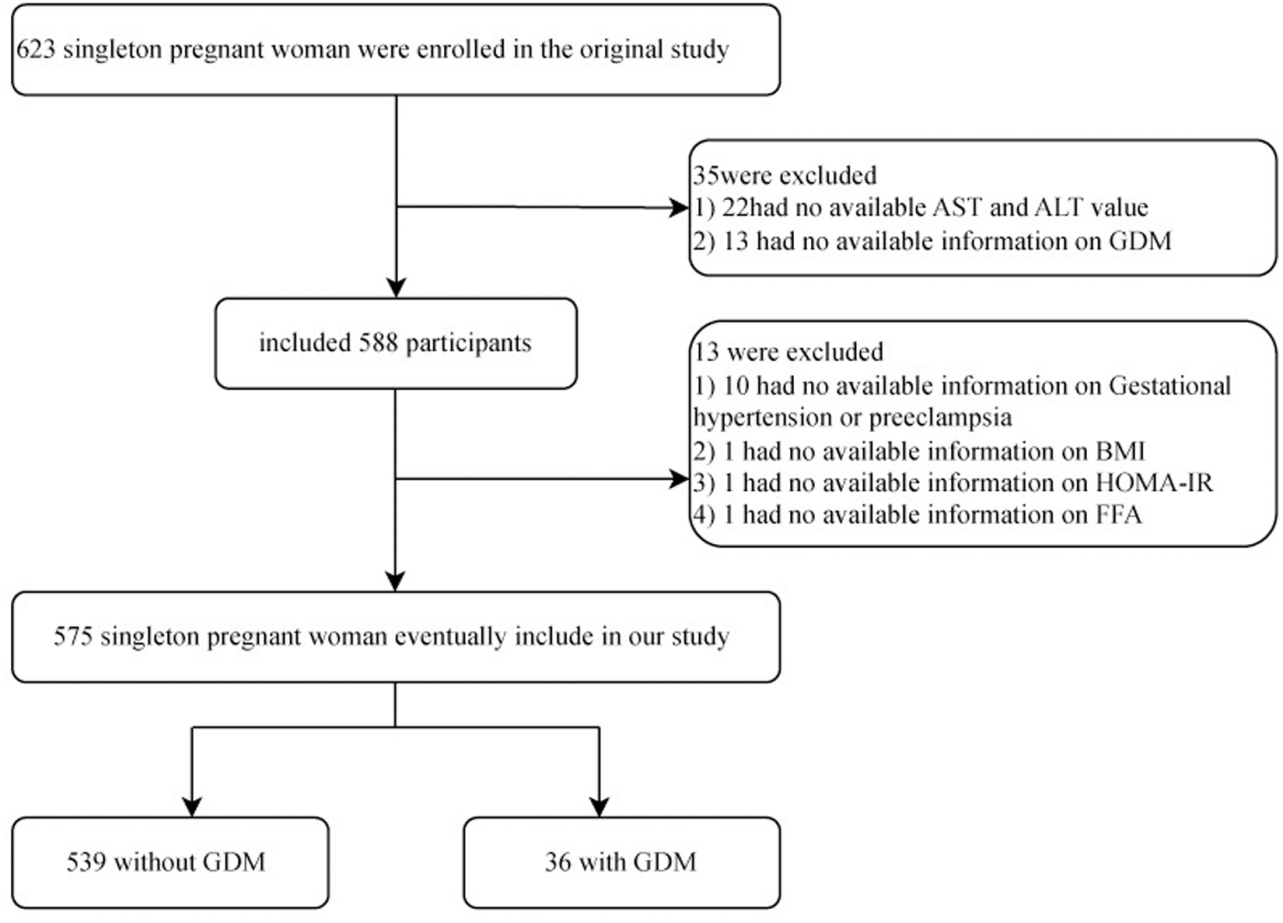
Flowchart of study participants.

The original study recruited singleton pregnant women attending labor and delivery before 14 weeks and included a total of 623 participants. Each participating pregnant woman signed an informed consent form. After our further screening, 48 participating pregnant women were excluded due to their absence of exposure variables (AST/ALT ratio, n = 22), outcome variables (GDM, n = 13), and covariates (n = 13) (Fig 1), and finally, we included a total of 575 participating pregnant women to assess the association of AST/ALT ratio with the incidence of gestational diabetes.

### Data collection and study design

Each pregnant woman was required to have her blood drawn between 10–14 weeks of gestation and fast for more than 8 hours at the time of blood draw. Blood tests were drawn for ALT, AST, total cholesterol (TC), triglyceride (TG), high-density lipoprotein (HDL) cholesterol, low-density lipoprotein (LDL) cholesterol, fasting glucose (FPG), insulin, and free fatty acids (FFA), while the pregnant women also had an ultrasound to assess the degree of liver fat. All participants were assessed for GDM by plasma glucose screening between 24–28 weeks of gestation [21]. Basic demographic and clinical information was also collected from each participant, such as age, whether they had ever given birth, whether they consumed alcohol, whether they had a history of liver disease, whether they had a history of diabetes mellitus, and height, weight, and blood pressure were checked by the staff, and the collection of basic data was carried out by professionally trained medical staff. Our study design was a prospective cohort study.

### Ethics statement

The initial ethical review of the study was approved by the Seoul National University Boramae Medical Centre Institutional Review Board and the Public Institutional Review Board of the Ministry of Health and Welfare of Korea. We are grateful to the researchers for their generosity in sharing their data. Since our study used public data for secondary analyses, no additional ethical approval was required.

### Exposure variables and outcome variable

The exposure variable was the AST/ALT ratio, obtained by dividing AST by ALT. GDM was diagnosed between 24 and 28 weeks of gestation, based on the results of an oral glucose tolerance test (OGTT), if the pregnant woman’s fasting plasma glucose reached or exceeded 5.1 mmol/L, or her plasma glucose reached or exceeded 10.0 mmol/L at 1 hour after sugar intake, or her plasma glucose reached or exceeded 8.5 mmol/L at 2 hours after sugar intake [22].

### Covariates

We used a comprehensive approach to identify risk factors associated with NAFLD from clinical expertise, original studies, and existing literature. Considering the above factors, covariates included age, previous birth experience, pre-pregnancy BMI, TG, TC, HDL-C, LDL-C, FFA, insulin, Hepatic steatosis, and insulin resistance index (HOMA-IR). HOME-IR is calculated as [FPG(mmol/Ltimesinsulin(μU/mL)/22.5] [23].

### Statistical analysis

Participating pregnant women were categorized into quartiles based on AST/ALT ratio: Q1 (<1.167), Q2 (1.167–1.499), Q3 (1.5–1.818), and Q4 (>1.818). Categorical variables were expressed as numbers and percentages and analyzed by chi-square test. Normally distributed continuous variables were expressed as mean and standard deviation and analyzed by ANOVA test. Continuous variables with skewed distribution were expressed as interquartile range and analyzed by the Kruskal-Wallis test.

Univariate and multivariate logistic regression analyses were used in this study. We developed four different models for multivariate logistic regression analysis (which is a statistical model that predicts the probability of an event occurring in a dichotomous problem). Model 1 was uncorrected for any factors and model 2 was corrected for socio-demographic characteristics such as history of childbearing and age. Model 3 was further corrected for BMI, TG, TC, HDL, LDL, and FFA, and model 4 was fully corrected to include insulin, Hepatic steatosis, and HOMA-IR. The study assessed the risk of GDM using adjusted odds ratios (OR) and a 95% confidence interval (CI) for the risk of GDM. The potential non-linear relationship between AST/ALT ratio and GDM was explored using restricted cubic spline (RCS) curves with full correction. Participants with BMI ≥ 25 were excluded from the first sensitivity analysis. Participants with NAFLD were excluded from the second analysis. We also used the AST/ALT ratio as a categorical variable, performed a logistic analysis of its association with GDM, and calculated *p*-values for trends to test the stability of the association between the AST/ALT ratio and GDM. Finally, stratified analyses were then performed, with stratified logistic regression analyses after grouping nulliparity, BMI, HOME-IR, and Hepatic steatosis.

We used the Free Statistical Analysis Platform (version 2.0, Beijing, China, http://www.clinicalscientists.cn/freestatistics) and the R statistical program (version 4.2.2, http://www.R-project.org, The R Foundation) for statistical analyses. *p* values < 0.05 were considered statistically significant, and the results were expressed as ratio ratios and 95% confidence intervals.

## Results

### Baseline characteristics of participating pregnant women

In this study, a total of 575 pregnant women were included. Table 1 describes the baseline characteristics of the participating pregnant women after quartiles according to AST/ALT ratio. The mean age was (32 ± 3.8) years. A total of 36 (6.3%) participants had GDM, of which 18 (12.9%) had GDM in group Q1, 6 (4.3%) had GDM in group Q2, 8 (5.3%) had GDM in group Q3, and 4 (2.8%) had GDM in group Q4. There was a significant difference in the incidence of GDM among the four groups, with group Q1 having a significantly higher incidence than the other three groups. The results in Table 1 indicate that group Q1 had higher BMI, TG, insulin, IR, hepatic steatosis, prevalence of GDM, and lower FFA. In addition to the marked differences between Q1 and other quartiles, our analysis revealed distinct metabolic patterns among Q2, Q3, and Q4. Q2 and Q3 exhibited significantly lower BMI and TG levels compared to Q1, with Q3 showing the lowest TG and highest FFA. Both Q2 and Q3 had lower HOMA-IR than Q1 and Q4, suggesting better insulin sensitivity.

**Table 1:** Baseline Characteristics of participants.

Characteristics	Total	Q1 (<1.167)	Q2 (1.167–1.499)	Q3 (1.5–1.818)	Q4 (>1.818)	*P* value
Age, Mean ± SD	32.0 ± 3.8	31.4 ± 3.8	32.2 ± 3.7	32.2 ± 3.9	32.4 ± 3.7	0.098
parity, n (%)						0.003
No	305 (53.0)	91 (65)	77 (55)	72 (47.7)	65 (45.1)	
Yes	270 (47.0)	49 (35)	63 (45)	79 (52.3)	79 (54.9)	
BMI (kg/m^2^)	22.0 ± 3.5	23.6 ± 4.0	22.0 ± 3.2	21.2 ± 3.0	21.4 ± 3.3	< 0.001
TG (mg/dL)	110.0 (87.0, 141.0)	123.0 (98.2, 158.0)	102.5 (83.2, 136.2)	99.0 (83.5, 125.5)	111.0 (88.0, 138.2)	< 0.001
TC (mg/dL)	172.6 ± 27.1	175.3 ± 26.5	173.2 ± 28.4	169.1 ± 26.0	173.3 ± 27.4	0.254
HDL (mg/dL)	64.8 ± 13.5	63.8 ± 13.7	65.1 ± 14.3	65.7 ± 12.7	64.6 ± 13.3	0.667
LDL (mg/dL)	83.9 ± 21.7	84.9 ± 22.1	85.3 ± 22.7	81.2 ± 21.0	84.5 ± 20.9	0.357
Hepatic steatosis, n (%)						0.011
Grade0	465 (80.9)	96 (68.6)	118 (84.3)	130 (86.1)	121 (84)	
Grade1	85 (14.8)	34 (24.3)	15 (10.7)	19 (12.6)	17 (11.8)	
Grade2	17 (3.0)	7 (5)	5 (3.6)	1 (0.7)	4 (2.8)	
Grade3	8 (1.4)	3 (2.1)	2 (1.4)	1 (0.7)	2 (1.4)	
FFA(μEq/L)	648.8 ± 272.3	593.2 ± 234.7	621.9 ± 232.8	698.8 ± 321.4	676.6 ± 275.5	0.003
FPG (mg/dL)	76.9 ± 9.8	76.3 ± 8.9	76.5 ± 11.7	77.2 ± 9.1	77.7 ± 9.0	0.643
NAFLD, n (%)						< 0.001
No	465 (80.9)	96 (68.6)	118 (84.3)	130 (86.1)	121 (84)	
Yes	110 (19.1)	44 (31.4)	22 (15.7)	21 (13.9)	23 (16)	
AST (IU/L)	17.8 ± 8.2	20.4 ± 7.8	16.6 ± 4.5	16.4 ± 3.9	18.1 ± 12.8	< 0.001
ALT (IU/L)	13.5 ± 9.7	24.3 ± 13.8	12.6 ± 3.5	10.1 ± 2.4	7.4 ± 2.5	< 0.001
Insulin (μIU/mL)	8.4 (5.3, 11.5)	9.9 (5.9, 14.4)	7.9 (5.2, 11.9)	7.4 (5.0, 10.9)	8.7 (6.0, 10.6)	0.008
HOMA-IR	1.5 (1.0, 2.3)	1.7 (1.0, 2.6)	1.4 (0.9, 2.3)	1.4 (0.9, 2.2)	1.7 (1.1, 2.2)	0.039
GDM, n (%)						0.002
No	539 (93.7)	122 (87.1)	134 (95.7)	143 (94.7)	140 (97.2)	
Yes	36 (6.3)	18 (12.9)	6 (4.3)	8 (5.3)	4 (2.8)	

parity(No: Never given birth; Yes: Have given birth); BMI: body mass index; TC, total cholesterol; TG, triglyceride; HDL, high-density lipoprotein cholesterol; LDL, low-density lipid cholesterol; FFA, free fatty acids; FPG, fasting plasma glucose; NAFLD, nonalcoholic fatty liver disease; AST, aspartate aminotransferase; ALT, alanine aminotransferase; HOMA-IR, homeostasis model assessment-insulin resistance; GDM, gestational diabetes mellitus.

### Relationship between AST/ALT ratio and GDM

**Fig 2:**
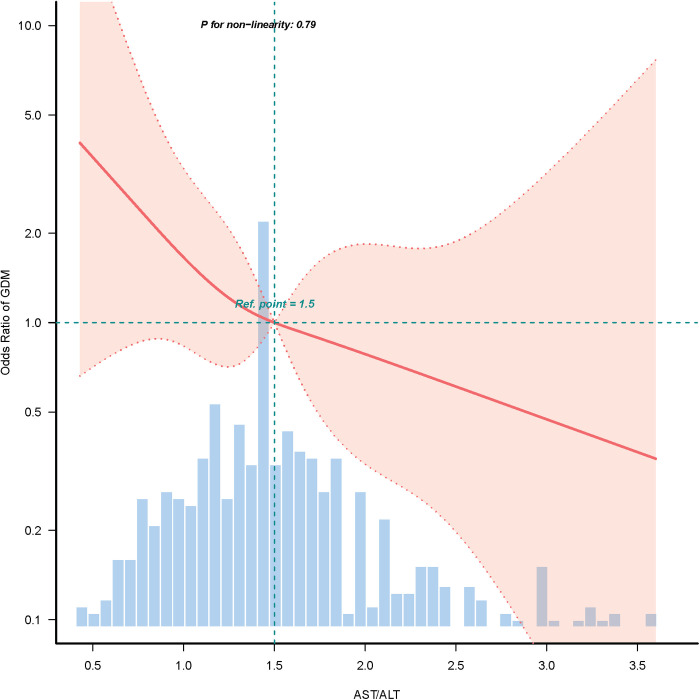
Association between AST/ALT ratio and GDM in RCS.

To further investigate the relationship between AST/ALT ratio and GDM, linear, logistic one-way, and multifactorial analyses were performed. As can be seen in Fig 2 even after adjusting for all covariates, the restricted cubic spline model still showed a linear relationship between AST/ALT ratio and GDM.

Univariate analysis of the occurrence of GDM (Table 2) showed that the risk of GDM was positively associated with BMI, ALT, TG, insulin, HOMA-IR, and hepatic steatosis and negatively associated with HDL and AST/ALT ratio.

**Table 2:** Univariate analysis of the occurrence of GDM.

Variable	OR-95 CI	*P*-value
Age	1.04 (0.95 ~ 1.14)	0.404
parity		
No	1	
Yes	1.01 (0.51 ~ 1.99)	0.974
BMI	1.27 (1.17 ~ 1.38)	<0.001
AST	1.02 (0.99 ~ 1.05)	0.185
ALT	1.04 (1.01 ~ 1.06)	0.002
TC	1.01 (1 ~ 1.02)	0.084
TG	1.02 (1.01 ~ 1.02)	<0.001
HDL	0.96 (0.94 ~ 0.99)	0.007
LDL	1 (0.98 ~ 1.02)	0.978
insulin	1.12 (1.07 ~ 1.17)	<0.001
AST/ALT	0.44 (0.22 ~ 0.9)	0.025
Hepatic steatosis		
Grade0	ref	
Grade1	3.32 (1.42 ~ 7.79)	0.006
Grade2	24.94 (8.51 ~ 73.09)	<0.001
Grade3	16.84 (3.7 ~ 76.66)	<0.001
HOMA-IR	1.47 (1.22 ~ 1.78)	<0.001
FFA	1.0012 (1.0001 ~ 1.0023)	0.032
FPG	1.0699 (1.0373 ~ 1.1035)	<0.001

Abbreviations: OR, odds ratio; CI, confidence interval

Multivariable logistic regression models analysis of the relationship between AST/ALT ratio and GDM in Korean Pregnant Women (Table 3) showed an independent negative association between AST/ALT ratio and the risk of GDM irrespective of any adjustment. In model 1, without adjusting for any covariates, each 1-unit increase in AST/ALT ratio was associated with a 56% reduction in the risk of GDM (95% CI: 0.22 to 0.9, *p* = 0.025). In model 2, after adjusting for age and parity, consistent results were obtained with a 58% reduction in GDM risk (95% CI: 0.2 to 0.87, *p* = 0.02). In model 3, after further adjustment for BMI, TG, TC, HDL, LDL, and FFA, the results remained significant, with a 55% reduction in the risk of GDM (95% CI: 0.21 to 0.98, **p* *= 0.044). In model 4, after continued adjustment for insulin, Hepatic steatosis, and HOMA-IR, the risk of GDM was reduced by 55% (95% CI: 0.19 to 0.97, **p* *= 0.043).

**Table 3:** Multivariable logistic regression models analysis of the relationship between AST/ALT ratio and GDM in Korean Pregnant Women.

Variable	Model 1		Model 2		Model 3		Model 4	
	OR (95%CI)	*P* value	OR (95%CI)	*P* value	OR (95%CI)	*P* value	OR (95%CI)	*P* value
AST/ALT	0.44 (0.22 ~ 0.9)	0.025	0.42 (0.2 ~ 0.87)	0.02	0.45 (0.21 ~ 0.98)	0.044	0.45 (0.2 ~ 0.99)	0.047
AST/ALT								
Q1 (<1.167)	1(Ref)		1(Ref)		1(Ref)		1(Ref)	
Q2 (1.167–1.499)	0.3 (0.12 ~ 0.79)	0.014	0.29 (0.11 ~ 0.75)	0.011	0.46 (0.15 ~ 1.38)	0.166	0.3 (0.08 ~ 1.12)	0.073
Q3 (1.5–1.818)	0.38 (0.16 ~ 0.9)	0.028	0.35 (0.15 ~ 0.85)	0.021	0.65 (0.21 ~ 2.02)	0.459	0.79 (0.25 ~ 2.5)	0.688
Q4 (>1.818)	0.19 (0.06 ~ 0.59)	0.004	0.18 (0.06 ~ 0.55)	0.003	0.2 (0.05 ~ 0.81)	0.024	0.16 (0.04 ~ 0.72)	0.016
P for Trend		0.002		0.001		0.034		0.035

Model 1 was adjusted for nothing.

Model 2 was adjusted for Age + parity

Model 3 was adjusted for Age + parity + BMI + TG + TC + HDL + LDL + FFA

Model 4 was adjusted for Age + parity + BMI + TG + TC + HDL + LDL + FFA + insulin + Hepatic steatosis + HOMA-IR

### Sensitivity analysis and subgroup analysis

**Fig 3:**
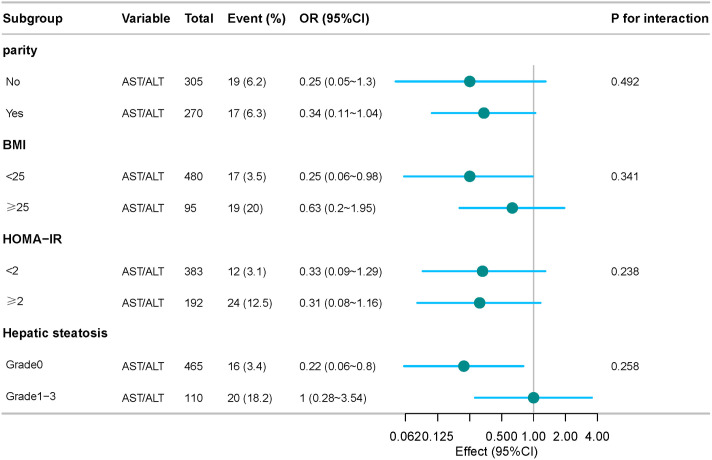
Forest plots of the size of the effect of AST/ALT ratio on GDM in prespecified and exploratory subgroups. In addition to the stratification factor itself, each stratification was adjusted for Age + parity + BMI + TG+ TC + HDL + LDL+ FFA + insulin + Hepatic steatosis + HOMA-IR.

Some studies have confirmed that GDM is associated with BMI and NAFLD, so we did two sensitivity analyses. After excluding pregnant women with BMI ≥ 25 kg/m^2^, the analyses were performed to show that the AST/ALT ratio was negatively associated with the risk of GDM after adjusting for all other covariates (OR: 0.25,95% CI: 0.06–0.98). Further, after we excluded patients with NAFLD, the AST/ALT ratio was still negatively associated with the risk of GDM in the fully adjusted model (OR: 0.22,95% CI: 0.06–0.8) (Table 4). After dividing the AST/ALT ratio as a categorical variable into four groups, the trend test showed that the linear relationship still existed. The correlation between AST/ALT ratio and GDM was consistent across subgroups as seen in the stratified analyses, and they remained robust regardless of parity, BMI, HOMA-IR, and hepatic steatosis (Fig 3). The consistency of this sample emphasizes the reliability of our results, suggesting that the relationship between AST/ALT ratio and GDM is not affected by these potentially confounding variables.

**Table 4:** Relationship between AST/ALT and GDM in different sensitivity analyses.

Variable	Model 1 (OR, 95% CI, *P*)	Model 2 (OR, 95% CI, *P*)
AST/ALT	0.25 (0.06 ~ 0.98) 0.047	0.22 (0.06 ~ 0.8)0.021

Model 1 was sensitivity analysis after excluding those with pre-pregnancy BMI ≥ 25 kg/m^2^, n = 480. We adjusted for Age, parity, TG, TC, HDL, LDL, FFA, insulin, Hepatic steatosis, and HOMA-IR.

Model 2 was a sensitivity analysis after excluding those with NAFLD, n = 465. We adjusted for Age, parity, BMI, TG, TC, HDL, LDL, FFA, insulin, and HOMA-IR.

## Discussion

This cohort study aimed to investigate the association between the first-trimester AST/ALT ratio and the incidence of GDM in Korean pregnant women. The study found that the AST/ALT ratio was negatively correlated with GDM, and the risk of GDM was reduced by 55% for each unit increase in the AST/ALT ratio.

In recent years, the prevalence of GDM in the general population in Korea has been increasing year by year, reaching 12.70% [24]. However, the incidence of GDM in this study was 6.26%, which is lower than in the literature. This is because our sample was screened from individuals without chronic liver disease, heavy alcohol consumption, and pre-pregnancy diabetes, all of which may have contributed to the lower incidence of GDM. Therefore, the incidence is within a reasonable range. GDM is associated with adverse pregnancy outcomes such as primary cesarean section, hypertensive disorders of pregnancy, and macrosomia [25]. Therefore, it is very important to find out the factors associated with GDM.

Numerous studies have demonstrated a link between ALT and AST with type 2 diabetes [26,27]. However, whether ALT and AST have an independent effect on GDM remains controversial. Although several studies have shown that there is a significant correlation between ALT and GDM, for example, in a study by Park, et. al, it was found that when blood ALT concentration increased to 17 U/L, it significantly increased the risk of GDM [28]. But still, there are more studies with opposite views on this, which concluded that there is no direct correlation between the two directly and the predictive value is limited [29,30]. High levels of AST may be associated with muscle damage and reflect metabolic abnormalities in the body, but its importance and reliability as an independent risk factor for predicting GDM is still subject to considerable debate [30]. Tan et al found no association between ALT and AST levels and GDM [31]. While another study showed that ALT levels were positively associated with GDM risk, there was no clear threshold [32]. Until recently, a large number of studies have suggested that the liver marker AST/ALT ratio can be considered as an indicator of IR [33,34], and is also associated with diabetes [35–37]. Meanwhile, Han et al. found that ALT/AST was associated with insulin resistance and metabolic syndrome in the Korean population [19]. Wang et al. found an inverse association between the AST/ALT ratio and the incidence of diabetes in a Chinese prediabetic population (HR = 0.40, 95% CI: 0.33–0.48, P < 0.001) [17]. In the study by Chen et al., the AST/ALT ratio was inversely associated with the risk of type 2 diabetes [18]. AST/ALT ratio was one of the best predictors of metabolic syndrome and T2 DM in Asian populations [38,39]. However, studies on the AST/ALT ratio and GDM are limited. We found a linear inverse association between AST/ALT ratio and GDM in a Korean pregnant population. At the same time, after we adjusted for BMI, IR, TC, TG, HDL, LDL, fatty liver degree, FFA, and other confounding factors related to GDM, the correlation still existed. Based on the above studies, we believe that the AST/ALT ratio is more important than using AST or ALT alone as an indicator of GDM. The AST/ALT ratio is more stable and independent, which can balance the fluctuation of the two and comprehensively reflect the degree of liver injury.

Some studies have demonstrated a positive correlation between the AST/ALT ratio and cardiovascular disease [40,41], whereas our study revealed an inverse association between the AST/ALT ratio and GDM. This discrepancy can be attributed to the distinct distribution patterns of AST and ALT in different tissues. AST is predominantly found in the liver, heart, and muscle tissue, whereas ALT is primarily localized in the liver. In cardiovascular diseases, myocardial cell injury leads to a significant increase in AST levels with minimal changes in ALT, resulting in an elevated AST/ALT ratio. Conversely, in liver-related conditions, both AST and ALT levels rise. However, since AST is mainly located in the mitochondria of hepatocytes and is released more slowly, while ALT is primarily distributed in the cytoplasm and is released more rapidly, the AST/ALT ratio tends to decrease in cases of liver injury. The AST/ALT ratio may exhibit a declining trend in cases of early liver injury. Although the mechanistic relationship between this ratio and GDM remains unclear, several plausible hypotheses have been proposed. First, the liver, as an important metabolic organ, plays an important role in the regulation of body sugar [42]. Impaired liver function affects the ability of the liver to store glucose after a meal, and diabetic patients often exhibit postprandial hyperglycemia and an inability to accumulate hepatic glycogen [43]. Studies by Martino et al. have shown that elevated ALT levels may be associated with weakened hepatic gluconeogenesis, which also means that a low AST/ALT ratio can cause abnormal glucose metabolism in the liver [44]. During pregnancy, the mother continuously supplies nutrients to the fetus, and we speculate that this process may lead to enhanced glycolysis as well as gluconeogenesis in the body, inhibiting hepatic glycogen synthesis and stimulating hepatic glucose production (HGP) in the body. It has been shown that activation of HGP leads to a series of altered transcription factor regulation, such as up-regulated expression of gluconeogenesis-related genes. In insulin clamp experiments, activation of HGP consistently stimulates insulin-mediated phosphorylation of the AKT signaling pathway, leading to alterations in cytosolic localization as well as in transcriptional activity of the corresponding regulatory genes, and reduced insulin sensitivity [45]. In addition, liver injury likewise affects the body’s fat regulation, and NAFLD is a common fatty liver-like disorder [46]. Studies have reported that the AST/ALT ratio might serve as a predictive marker for the occurrence of NAFLD [47]. Meanwhile, the occurrence of NAFLD is also closely associated with IR in the body, so we also speculate that IR might be another mechanism. Furthermore, studies have shown that hepatocyte injury triggers the release of hepatokines, which can induce peripheral insulin resistance by interfering with insulin signaling in skeletal muscle and adipose tissue [48,49]. This creates a vicious cycle where hepatic insulin resistance begets systemic insulin resistance, further exacerbating glucose intolerance. The low AST/ALT ratio may serve as a marker for this pathological cascade, as it reflects both the initial hepatic steatosis and the subsequent mild hepatocyte injury. The link between low AST/ALT ratio and GDM risk is further supported by emerging evidence on the role of hepatic mitochondrial dysfunction in glucose metabolism. In states of hepatic steatosis, mitochondrial β-oxidation becomes impaired, leading to the accumulation of reactive oxygen species and the subsequent activation of stress kinases at inhibitory sites [50]. This molecular mechanism provides a direct pathway from hepatic lipid accumulation (reflected by low AST/ALT) to impaired insulin signaling and glucose dysregulation. In conclusion, we believe that GDM is mainly associated with insulin resistance during pregnancy, and the low ratio of AST/ALT may reflect the predisposition of the liver to low insulin sensitivity at this stage due to insufficient metabolic demand as well as mild injury.

The present study has the following strengths. Firstly, we employed the AST/ALT ratio as both a continuous and categorical variable, thereby demonstrating that the AST/ALT ratio is independently and negatively associated with GDM when Korean pregnant women are considered as the study population. Secondly, we adjusted for confounders as much as possible. Thirdly, we performed sensitivity analyses to exclude individuals with BMI ≥ 25 and NAFLD before performing the association between AST/ALT ratio and GDM, respectively. Subgroup analyses further confirmed the study’s stability.

This study has several limitations. First, a single measurement of the AST/ALT ratio at 10 ~ 14 weeks of gestation cannot account for potential physiological fluctuations during pregnancy, additional measurements during mid- and late-pregnancy would help assess temporal variability and the robustness of the association. Second, although we corrected for known major confounders, residual confounders from unmeasured variables such as dietary factors, physical activity, and genetic predisposition may have an impact on the results. As we were a secondary analysis, there was no control over the collection of the original data. In future studies, we need to incorporate as many factors as possible, such as diet, physical activity, and genetics. Third, with only 36 GDM cases, the power to detect subtle associations may be limited. Larger cohorts could validate the findings. At the same time, the population of our study is Korean, and it is necessary to expand the study to other races and populations to increase the general adaptability of the study. Fourth, because this study was a secondary analysis, the original study did not exclude participants with heart disease, and the changes in AST may have been cardiac-related. Future studies of ours will need to exclude participants with heart disease. Finally, although we observed a significant negative correlation between the AST/ALT ratio and the risk of GDM, the underlying biological mechanisms remain speculative and need to be experimentally validated by animal models or in vitro studies.

Although our study suggests that the AST/ALT ratio in early pregnancy is an independent predictor of GDM, contemporary clinical decision-making increasingly favors comprehensive risk-stratification models that combine biomarkers and clinical parameters. To improve clinical applicability, future studies should explore the incorporation of the AST/ALT ratio into a multifactorial risk scoring system similar to the Cardiology Framingham Risk Score [51]. Specifically, a comprehensive GDM risk score could combine the AST/ALT ratio with existing predictors (e.g., maternal age, pre-pregnancy BMI, family history of diabetes mellitus, fasting blood glucose level, and inflammatory markers) via a weighting algorithm or machine learning approach. In addition, continuous monitoring of the AST/ALT ratio during pregnancy may provide a dynamic risk-assessment function that may enable early intervention. Our study focused on the Korean population, where the available literature suggests that the prevalence of GDM is approximately 11.1%, which is lower than in many Western and Asian countries, such as Australia (14%) and Saudi Arabia (49.5%) [2]. This difference is due to factors such as diagnostic criteria, demographics, lifestyle, and underlying genetic susceptibility. Because of the differences in these factors, extrapolation of the findings to other ethnic groups needs to be done with caution, and future comparative studies in different populations are needed.

## Conclusion

In conclusion, our study showed that the AST/ALT ratio was independently and negatively associated with GDM in Korean pregnant women and that the probability of GDM was higher in those with a low AST/ALT ratio.
